# Clinical audit of cases and outcomes of patients admitted to the intensive care unit at Kamuzu Central Hospital, Lilongwe, Malawi

**DOI:** 10.1038/s41598-024-66810-7

**Published:** 2024-08-16

**Authors:** Akim Nelson Bwanali, Leonard Munthali, Upile Napolo, Adriano Focus Lubanga, Rodwell Gundo, Samuel L. Mpinganjira

**Affiliations:** 1 School of Global and Public Health, Kamuzu University of Health Sciences, Blantyre, Malawi; 2 School of Nursing, Kamuzu University of Health Sciences, Blantyre, Malawi

**Keywords:** Critical care, Intensive care unit (ICU), Malawi, Diseases, Health care

## Abstract

In 2016, a new, improved and modern intensive care unit was constructed at Kamuzu Central Hospital in Lilongwe, Malawi. Having been operational for about 4 years, there has not been a systematic audit to gauge its performance. Therefore, this quantitative retrospective cohort study aimed at investigating the performance of the intensive care unit at Kamuzu Central Hospital in Lilongwe, Malawi. We analysed the patterns of admission through 250 clinical cases and their respective outcomes spanning from 1st January 2019 to 31st December 2019 using STATA. Descriptive and inferential statistics were computed. We also had a follow-up discussion with the Head of the unit to better understand the unit’s functioning. Out of the 250 admissions, we evaluated 249 case files. About 30.8% of all patients were referred from the main operating theatre, and 20.7% from the casualty (emergency medicine). Head injury (26.7%) and peritonitis (15.7%) were the commonest causes of admission. The overall mortality was 52.2% with more females (57.5%) dying than males (47.9%). Head injury and peritonitis had the highest contribution to the mortality accounting for 25.3% and 16.9% of all deaths respectively. In conclusion, despite the new unit registering an improved performance compared to the old unit’s 2012 mortality of 60.9%, the current mortality rate of 52.2% generally reflects a suboptimal performance. The intensive care unit is still grappling with a number of challenges that need immediate attention including few working beds, shortage of critical care specialists and nursing staff and lack of standard admission criteria.

## Introduction

An Intensive Care Unit (ICU) is an organized system for the provision of care to critically ill patients that provides intensive and specialized medical and nursing care. It harbours enhanced capacity for monitoring, and multiple techniques for physiologic organ support such as mechanical ventilation to sustain life during a period of life-threatening organ system insufficiency^1^.

To function optimally, such an ICU must be complemented with a good infrastructure, consistent supply of drugs, advanced medical equipment and well trained personnel^2^. Since the onset of critical care provision, the global community has witnessed dramatic improvements in recovery of those with critical illnesses and injuries^3^. This is precisely true for developed countries whose resources enable ICUs to function optimally^4^.

Developing countries, in contrast, often fail to operationalize the same high quality of care^3–5^. In sub-Saharan Africa (SSA), ICUs often run on limited equipment and healthcare providers usually with no specialized training in critical care^5^. These differences on the quality of critical care provision between developed and developing countries have led to profound differences. Not surprising, their ICUs register mortality rates as low as 8% in comparison to 64% as reported by other low-income country studies^6–8^. Unfortunately there is limited data on impact that subpar critical care is having on the performance of the whole health sector^9^.

Mortality and outcomes of ICU admission have been variable across studies in Africa. For instance, a study in Uganda found an overall mortality of 25% in comparison to 41% found in Tanzania^3,10^. Multiple studies in SSA have reported numerous factors associated with poor outcomes in ICU which include advanced age, urgency of admission and development of in-hospital complications^11–14^.

The challenges and poor critical care performance highlighted above could be heightened in Malawi due to an especially increased demand of critical care to provide for the rapidly growing population, thereby placing a further burden on the already strained healthcare workforce. However, there is limited local data on factors that influence outcome of patients admitted to the ICU in Malawi.

Malawi built her first 5-bed ICU in 1990, at Kamuzu Central Hospital (KCH)^15^. In 2016, the ICU at KCH was transferred to a new building with more space, beds and equipment, to cater for the population of the central region. This new ICU has 8 beds with 1 presidential suite bed. A previous study conducted in the old ICU at KCH showed that the ICU admits an average of 22 patients per month with 5 functional beds a time due to limited staff. Alarmingly, one study in 2012 at KCH ICU revealed a general mortality of 60.9% with sepsis as the commonest causes of death^16^. Since commencement of the newly constructed ICU there is no systemic study done to assess its performance and ascertain any improvement from the old ICU. Therefore, we aimed to assess clinical outcomes of patients, commonest causes of mortality for patients admitted at KCH ICU and compare the performance of the new ICU with the old one.

## Materials and methods

### Study setting

This study was conducted at KCH newly-built ICU. KCH has an 8-bed capacity ICU with only 5 operational beds to serve a population of 7,523,340 in the central region of Malawi^17^. This creates a ratio 1 bed available for every 1.5 million people. The ratio of the ICU beds to the total number of hospital beds at is 1:150. The unit is currently being run by the head of the anaesthesia department. However, there is no intensivist. At any point in time, there is a clinician/anaesthetist on-call and 5 nurses on-duty. Thus a nurse to patient ratio of 1:1 is usually maintained. Among the 21 nurses allocated to the ICU, only one of them underwent special critical care training. Additionally, there is no established team that carries out ward rounds. As such, the referring units periodically come to evaluate their respective patients.

The ICU is for both paediatric and adult patients. Each ICU bed has a ventilator, perfuser, an infusion pump, a multi-parameter monitor and an oxygen concentrator. A defibrillator and a 12-lead ECG are available for use at any bed needed. However, the ICU does not have the following; a portable X-ray, an ultrasound machine and an arterial blood gas analyser.

### Study design

This was a quantitative retrospective review conducted at KCH ICU. We aimed to review 250 case files of patients ever admitted in this ICU between January 2019 and December 2019. They were conveniently sampled.

### Data collection

All data was collected by the primary investigators for the study (ANB, LM and UN). The data collection period span from 1 September, 2021 to 14 September, 2021.We collected data from the ICU register book onto a case reporting form. Variables included patient’s code number (that we assigned), referring unit, age, sex, diagnosis, interventions done, length of admission and their outcome. The length of stay in the ICU was measured in calendar days based on the admission and discharge dates. Additionally, we held a follow-up discussion with the Head of Unit to gain further insights into the functioning of the unit.

### Data management and analysis

The data was then cleaned and transferred into an Excel 2016 electronic file which was kept on a password-secured personal laptop and backed up on a secure online drive.

We employed descriptive statistics to describe our sample. Categorical variables were summarized into frequencies and percentages and presented in frequency distribution tables, graphs and pie charts. Continuous data was summarized as means, standard deviations and medians and further summarized and displayed into frequency polygons/histogram.

Comparison of categorical variables was performed using Chi squared test. Correlation between the variables were tested using the Pearson correlation co-efficient. A *P*-value of less that 0.05 was considered significant. Statistical analyses were conducted using STATA 16.

### Ethical considerations

The approval to conduct the study was obtained from Kamuzu University of Health Sciences (KUHES) research and ethics committee, reference number U.03/20/3027. Further approval was obtained from the hospital director and the Head of ICU at KCH. The study was conducted in accordance to the ethical rules and regulation as stipulated in the KUHES ethics guidelines. The need of informed consent was waived by KUHES research ethics since the study did not involve any physical interaction with the patient. The study has no anticipated risk, as it involved secondary analysis of routinely collected data. To ensure confidentiality and anonymity, we de-identified and disaggregated the data. Only the primary researchers accessed the data.

## Results

### Demographic profile

A total of 250 case files for patients of all age groups admitted to the ICU in a period spanning from January 1st, 2019 to December 31st, 2019 were reviewed. Males made up 57% of the cases (Table 1). The age of the patients ranged from less than 1 to 87 with a mean of 30 ± 18.4 years and had a mean length of admission of 4.93 ± 5.06 days.

**Table 1:** Patient characteristics and outcomes.

Variable	Died (%)	Survived (%)	*P* Value
All admissions	130 (52.2)	119 (47.8)	
Sex (n = 249)			0.132
Male	68 (47.9)	74 (52.1)	
Female	61 (57.0)	46 (43.0)	
Age in years (n = 248)			0.067
Under 5	14 (58.3)	10 (41.7)	
5–17	20 (62.5)	12 (37.5)	
18–29	29 (40.3)	43 (59.7)	
30–49	39 (50.6)	38 (49.4)	
Above 50	28 (65.1)	15 (34.9)	
Diagnosis (n = 247)			0.418
Head injury	33 (50.0)	33 (50.0)	
Peritonitis	22 (56.4)	17 (43.6)	
Septic shock	13 (56.5)	10 (43.5)	
Post-operative complications	6 (33.3)	12 (66.7)	
Poly trauma	10 (58.8)	7 (41.2)	
Respiratory distress	7 (50.0)	7 (50.0)	
Post-partum haemorrhage	5 (55.6)	4 (44.4)	
Eclampsia, pre-eclampsia	5 (55.6)	4 (44.4)	
Pulmonary oedema	6 (75.0)	2 (25.0)	
Severe pneumonia	4 (50.0)	4 (50.0)	
Visceral rupture/injury	4 (50.0)	4 (50.0)	
Cardiac arrest	4 (80.0)	1 (20.0)	
Chest injury	2 (66.7)	1 (33.3)	
Poisoning	0 (0.0)	3 (100.0)	
Organ failure	2 (66.7)	1 (33.3)	
Burns	2 (100.0)	0 (0.0)	
Other	5 (41.7)	0 (0.0)	

Of the 250 cases, some patients had missing variables. 0.4% were missing sex, another 0.4% had no outcome recorded, 0.8% had undocumented age, 1.2% did not have referring unit and another 1.2% did not have a diagnosis.

**Figure 1:**
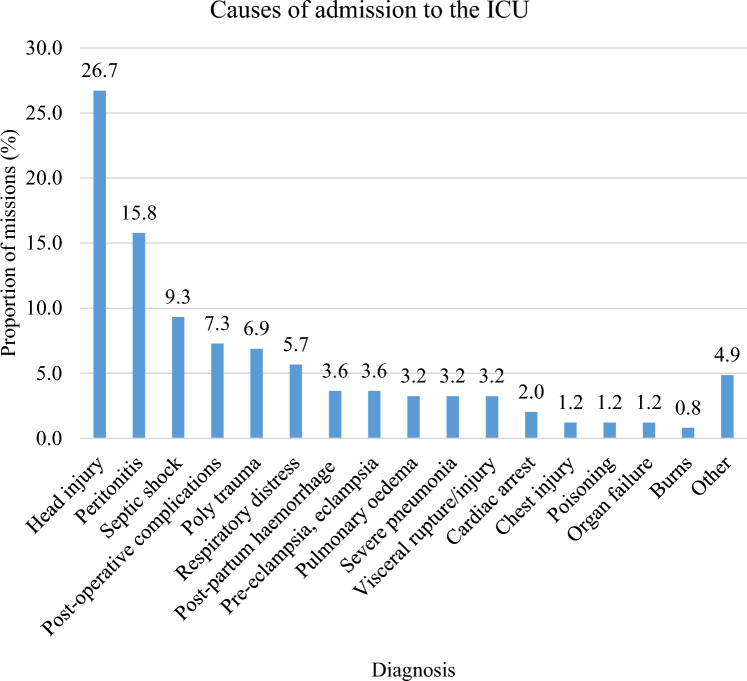
Causes of admission to the ICU. Head injury (26.7%) and peritonitis (15.8) were the commonest causes of admission to the ICU.

**Figure 2:**
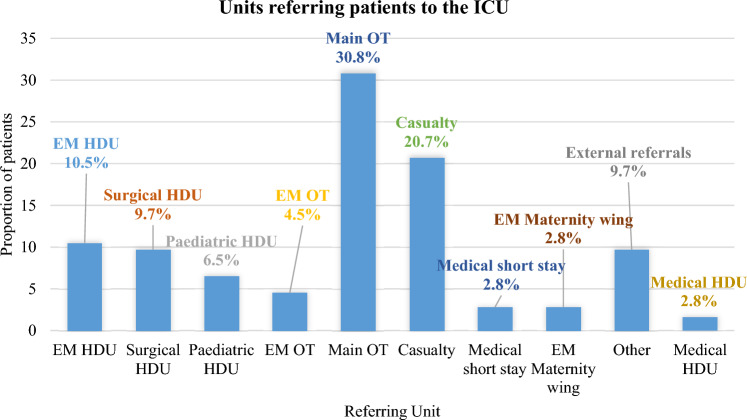
Relative contributions of various referring units to the ICU admissions. Most patients were referred from the main operating theatre and casualty (emergency medicine department). Key: Ethel Mutharika; HDU, High Dependency Unit; OT, Operating theatre.

**Figure 3:**
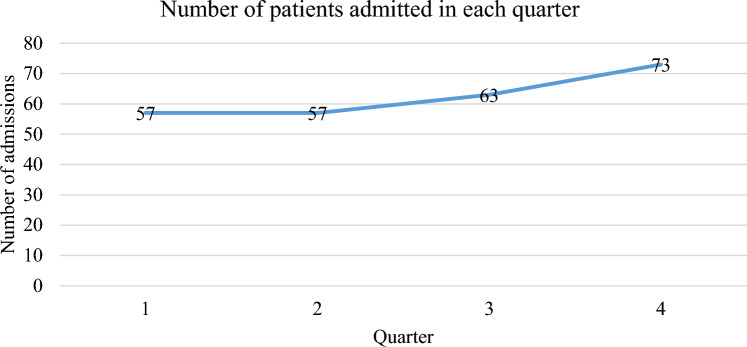
Number of admissions in quarters of the year. Most admissions took place in the third and fourth quarters of the year 2019.

The commonest diagnoses at admission were head injury and peritonitis which contributed 26.7% and 15.7% respectively (Fig. 1). Most of the patients were referred from the main operating theatre (30.8%), followed by the Casualty (20.7%) and the Ethel Mutharika (EM) Maternity High Dependency Unit (HDU) (10.5%) (Fig. 2). Most of the patients required mechanical ventilation (90.8%). A smaller proportion were given catecholamines (24.8%) and an even smaller proportion underwent Cardiopulmonary Resuscitation (CPR) (2.4%). Over half of the admissions took place in the second half of the year (54.4%) (Fig. 3).

### Treatment outcomes

**Figure 4:**
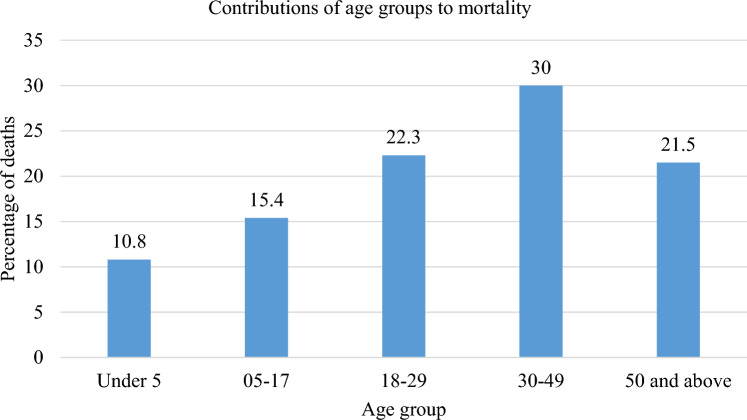
Composition of age groups in the overall mortality. Most deaths were contributed by the 30–49 age group (30%). The paediatric population (children) contributed a combined quarter of the mortality.

The study revealed an in-ICU mortality rate of 52%. The mortality was higher amongst females with a mortality rate of 57.5% (compared to 47.9% in males) (Table 1). Cardiac arrest and pulmonary oedema were conditions with the highest in-ICU mortality of over 70% each. However, head injury and peritonitis had the highest contribution to the mortality accounting for 25.3% and 16.9% of all deaths respectively. The highest mortality rate was observed in those older than 50 years (65.1%) and the paediatric group (62.5%) (Fig. 4).

### Follow-up discussion with the head of unit

It transpired that the unit operates on substandard admission criteria that is not supported by any disease severity and outcome prediction scoring systems. Admission of patients into the unit is mainly based on the admitting officer’s clinical judgement, resulting in subjective admission of patients into the ICU.

## Discussion

This study aimed at bringing to light the performance of the ICU. Despite the new ICU registering an improved performance compared to the old ICU, the mortality rate of 52.2% generally reflects a suboptimal performance compared to other ICUs within the SSA.

Our study portrayed inadequacy of ICU beds with a shocking ratio of only 1 bed available for every 1.5 million people. Another study done at another tertiary hospital in the same country showed a similar ratio^6^. Elsewhere, in Uganda and other parts of Africa, the ratio still stands at 1 to 1 million^5^. This is in massive contrast to Western countries. For example, there is 1 bed for every 293 people in the USA and 1 for every 163 people in Germany. Surprisingly, the demand for critical care services is arguably more enormous in sub-Saharan Africa. This is because the high incidence of road traffic accidents and infectious diseases in developing countries places an additional burden on critical care services^18^. Sadly, the discipline of critical care is heavily eluded from priority in these settings as it gets minute proportion of the health sector funding^4^. Sufficient resource allocation to the specialty of critical care is therefore required to improve provision of care to the critically ill and their overall prognosis.

The results of our study portrayed head trauma to be the most common diagnosis within the ICU with a total percentage of 26.7%, of cases of which 86% of the head injury patients were males. This could be attributed to a rising incidence of road traffic accidents (RTAs). A study conducted in 2017 showed that there has been a rapidly growing burden of RTAs at KCH in Lilongwe with a prediction that the burden will double by 2030^18^. 76.4% of those with road traffic injuries were males with an average age of 24.2. With young productive males losing days at work and their lives due to devastating injuries, this leads to a reduction in the productive population of the nation. This ultimately affects the economy. It is said that in Malawi there is an estimated impact of 5% to the GDP^18^. This therefore, calls for a multi-sectoral approach to address the key causes of admission to the ICU including ensuring road traffic safety.

Our study revealed an overall mortality of 52.2%. A study conducted in 2012 at KCH’s previous ICU found an overall mortality of 60.9%, thus signifying a reduction of approximately of 8% with the new ICU^16^. This may be attributed to the increase in resources. However, a study conducted at another tertiary level hospital Queen Elizabeth Central Hospital (QECH) displayed an overall mortality was 23.6% which is significantly lower than that of KCH although QECH had a lower bed capacity^6^. The differences may be explained by the fact that at KCH’s ICU patients are not attended by physicians on a daily basis. Additionally, the lack of a standard ICU admission criteria means some patients are admitted in a moribund state, which only serves to increase the overall mortality. Another study in South Africa showed an overall mortality of 13.6%, which is another alarming difference^19^. That study ICU has 16 beds and full time specialist cover which seems to make a big difference to the outcome of patients. Of note is that they also use a scoring system known as the Simplified Acute Physiology Score (SAPS 3) to assess severity of patients presenting to the ICU. The scoring system helps predict the realistic outcome of the patients. In contrast, at KCH the admission criteria does not have any scoring system (mainly based on admitting officer’s clinical judgement) resulting in subjective admission of patients into the ICU. We should therefore have an anaesthesiologist to do daily ward rounds on patients and implement an ICU scoring system.

This study revealed a mortality rate of up to 62.5% among the paediatric patients admitted in the ICU. In a study done at another tertiary hospital in the same country, the paediatric mortality rate was 32%^6^. These findings are comparable to similar studies done across Africa. The mortality rates reported lie within the range 20–60%^20,21^. This is alarming, considering the fact that children comprise a quarter or less of the ICU patients. Interestingly, some studies done in resource-rich settings have demonstrated better outcomes in critically ill children managed in dedicated Paediatric Intensive Care Unit (PICU) as compared to those managed in general ICU^22^. However, studies in dedicated PICU in Rwanda and South Africa contradict such a finding with mortality rates of 50% and 56% respectively^23^. This highlights the general subpar provision of critical care in developing countries. Keys to improving the overall intensive care provision could prove useful in reducing child mortality during care.

## Conclusion

Our study established that critical care medicine is still underdeveloped in low resource settings. For Malawi, it showed that over 50% of the patients die. Most of the patients were admitted due to surgical conditions with head injury and peritonitis being the commonest causes of admission and head injury contributing the highest to mortality. Most of the patients were referred from the main operating theatre.

## Recommendations

The study recommends provision of medical and nursing staff with adequate training and expertize to provide critical care medicine. The study and other similar studies across SSA persistently highlight the key challenge of inadequate staffing contributing to high mortality rates among the critically ill patients. This requires raising political commitment toward critical care by increasing financial resources allocated to the specialty which is currently neglected.

Furthermore, it is crucial to develop and utilise an evidence-based and standardized ICU admission criteria. As our study has reported, most patients are admitted based on the admitting officer’s clinical judgement resulting in admitting patients who may have little chance of benefitting from critical care. There is therefore, a need to establish legitimate admission criteria to ensure that the limited ICU resources are used on patients with the best possible chance of benefitting from critical care.

Finally, we also recommend an improved electronic documentation system for the provision of ICU care. This could ensure an effective evaluation of the performance of the ICU.

## Study limitations

Some of the required data about the patients was missing from the registry. This just goes to show how poor the level of documentation within the hospital is. Secondly, we were unable to determine the exact causes of death due to unavailability of autopsy data. Furthermore, we were unable to follow up on the patients that were transferred out, to determine their survival rate as their files were not in the ICU. Additionally, due to the lack of a clear admission criteria or scoring system, we do not know which patients truly qualified for ICU care. Last but not least, data was only collected for the year 2019 despite the new ICU being operational for 4 years hence the results are subject to confounding factors associated with the year 2019. Besides, the study was conducted at one hospital, hence, generalization of the findings is limited.

## Data Availability

The data obtained and/or analysed during the study is available and can be provided upon a reasonable request made to the corresponding author.
